# Placebo Effects on Stress, but Not on Pain Reports. A Multi-Experiment Study

**DOI:** 10.3389/fpsyg.2021.639236

**Published:** 2021-06-07

**Authors:** Sara Magelssen Vambheim, Hojjat Daniali, Magne Arve Flaten

**Affiliations:** ^1^Department of Pain Management and Research, Oslo University Hospital, Ullevål, Norway; ^2^Department of Psychology, Norwegian University of Science and Technology, Trondheim, Norway

**Keywords:** placebo effect, placebo response, experimenter sex, participant sex, negative emotions, stress, blood pressure

## Abstract

**Background:**

Contextual factors, such as participant/experimenter sex may moderate the placebo effects. We tested whether the participant and experimenter sex modulated placebo effects on experimentally induced pain and associated stress.

**Objective:**

To investigate if (i) participant sex and (ii) experimenter sex influence placebo analgesia and subjective and physiological stress in two experiments employing a within-subjects and a mixed design, respectively. Placebo effects were investigated in pain reports, stress, and blood pressure.

**Methods:**

Participants received painful stimulations and a placebo cream. In *Experiment One* (*N* = 59) participants underwent a placebo condition (PC) and a natural history condition (NHC) in random order. A placebo cream was applied in the PC and then the heat stimulation temperature was surreptitiously lowered. Identical stimulations were administered in the NHC, but with no cream, no information, and no lowered temperature. In *Experiment Two*, participants (*N* = 93) were randomly assigned to three groups receiving either a placebo cream with surreptitiously lowered intensity of electric stimuli (Placebo, PG), a placebo cream (Cream-Control, CCG) without changing the stimuli, or lowered intensity, but with no cream (Pain-Control, PCG) in a mixed design. All participants in both experiments received the same stimuli in the post-test as in the pre-test. Four experimenters (two females) in *Experiment One*, and five experimenters (two females) in *Experiment Two* conducted the studies.

**Results:**

No placebo effect was seen on pain. However, there were placebo effects on stress, moderated by participant and experimenter sex: in *Experiment One* males in the PC had lower diastolic blood pressure (DBP) compared to males in the NHC. Participants in the PC had lower DBP compared to the NHC when tested by a female. In *Experiment Two*, participants expected more cream effectiveness when a female experimenter administered it, and reported lower stress in the PG compared to the PCG when tested by females.

**Conclusion:**

Our findings highlight a distinction between placebo effects on pain and on associated stress. Secondly, female experimenters recorded lower physiological and subjective stress, higher effectiveness expectations, and lower pain from both sexes compared to male experimenters. Possible reasons for the failure to find a pain placebo effect are discussed.

## Introduction

A placebo effect is a reduction in pain and other symptoms following the administration of an inert element introduced as an effective treatment or previously experienced as so. Placebo effects are mostly due to positive expectations of treatment efficacy (e.g., Bjørkedal and Flaten, 2012) initiated by verbal information (Flaten et al., 2006) and/or learning processes (e.g., Colloca and Benedetti, 2009; Flaten et al., 2013; Bajcar and Bąbel, 2018; Bąbel, 2020). Verbal information about the effect of a treatment, e.g., telling the participant that a capsule will effectively reduce pain, has been shown to reduce pain (Flaten et al., 1999). Classical conditioning is based on a personal experience that a treatment/manipulation has reduced a symptom e.g., pain. The effect of the treatment is the unconditioned stimulus (US) and the reduced pain is the unconditioned response (UR), and other stimuli like the shape, taste or color of the capsule may become conditioned stimuli (CS) that elicit a conditioned response (CR) of reduced pain (e.g., Montgomery and Kirsch, 1997).

However, placebo manipulations, as in other treatments, are inevitably administered along with a compound of other contextual (e.g., treatment setting, medication features; Di Blasi et al., 2001), interrelationship (e.g., experimenter sex; Aslaksen and Flaten, 2008; Daniali and Flaten, 2019) and individual factors (e.g., participant sex; Vambheim and Flaten, 2017) which are capable of modulating the placebo effects and in a larger context, treatment outcomes.

The term sex refers to biological differences between males and females. On the other hand, gender refers to a set of expectations that a society attributes to each sex (Sanford et al., 2002) from early childhood (Atkinson and Endsley, 1976). Respecting pain behavior, western societies expect the male character to be pain tolerant, whereas the female character is expected to be pain sensitive and seek protection from males (Sanford et al., 2002). Such characteristics can modulate pain report and placebo effects. For instance, Vambheim and Flaten (2017) reported that males are more responsive to placebo treatment, whereas females are more responsive to nocebo treatment. Recently, Enck and Klosterhalfen (2019) showed that males are more responsive to verbally generated placebo treatments, while females are more responsive to placebo treatments generated by classical conditioning. However, the inconsistent findings (e.g., females also have been shown to be more responsive to verbally produced placebo effects; Olson et al., 2021), and the interaction of participant and experimenter/clinician sex (e.g., Mogil, 2012), have made it difficult to understand the effects of sex/gender on placebo effects.

As mentioned, the characteristics of the observer, such as the experimenter/clinician sex may activate gender role expectations in the person experiencing the pain, and therefore affect pain report and placebo effects. For instance, Flaten et al. (2006) showed that male participants reported lower pain and higher placebo effects to female experimenters and concluded that this was due to a bias in males to report less pain to female experimenters. In the same line, Daniali and Flaten (2019) reported that experimenters received lower pain reports from the participant of the opposite sex. However, very few studies have investigated the effects of experimenter/clinician sex on pain and placebo effects, but with incompatible findings (e.g., Daniali and Flaten, 2019).

Moreover, participant and experimenter sex can also affect negative emotions associated with pain or other noxious stimuli, even though very few studies have suggested a mediating role for participants’ sex on placebo responses on emotions associated with pain (e.g., Colloca et al., 2016) or with other symptoms (e.g., Abrams and Kushner, 2004). Colloca et al. (2016) reported that, compared to male participants, females had higher placebo responses and lower physiological stress, as indexed by cortisol levels. Abrams and Kushner (2004) reported that males with higher expectations that an alcoholic drink would reduce their tension, had lower anxiety following the consumption of a placebo beverage compared to females. Apparently, the mediating effect of participant sex on placebo effects have been reported mostly on pain, and less on stress associated with pain. Therefore, there is little information on how participants’ sex modulates negative emotions associated with pain. Except Aslaksen and Flaten (2008) that found no effects of experimenter sex other than on pain reports, no research has reported effects of experimenter sex on stress associated with pain. As suggested by Flaten et al. (2011), placebo analgesic effects are partly due to a reduction in stress. Thus, if females displayed larger placebo responses after a conditioning procedure, this should be accompanied by a reduction in physiological and subjective stress (see also Lyby et al., 2011).

Therefore, two experiments were carried out to investigate the effects of participant and experimenter sex on placebo effects/responses on experimentally induced pain. We chose to consistently use the term sex instead of gender for all measures of differences between males and females, due to inclusion of both physiological and psychological measurements. As suggested by Enck and Klosterhalfen (2019), both classical conditioning and suggestive verbal information were used to generate placebo effects in both experiments.

We chose the main outcome variables to be subjective pain reports (pain intensity and unpleasantness; see methods) due to the extensive literature supporting their use in laboratory and clinical studies (e.g., Al’Absi and Flaten, 2016). We also explored whether participant and experimenter sex moderated subjective and physiological stress levels (i.e., blood pressure) associated with pain. Pain can act as a stressor and alter the autonomic nervous system (ANS) activity (Craig, 2003). As cardiovascular activity is controlled by the ANS, measuring cardiac activity can indicate corresponding psychophysiological changes due to the stress induced by pain (Loggia et al., 2011). In this study we used blood pressure (BP) as a physiological stress index. BP has been used as an index of physiological stress in former studies with similar aims (e.g., Geers et al., 2015; for a review see Daniali and Flaten, 2020).

Therefore, the following main hypotheses were tested: (a) compared to males, female participants should have higher placebo effects (i.e., lower pain reports); (b) participants should have higher placebo effect (i.e., lower pain report) to experimenters of the opposite sex. (c) Following the placebo manipulations, female participants should have lower subjective and physiological stress levels compared to males; and (d) following the placebo manipulations, participants should have lower subjective and physiological stress levels when being tested by female experimenters.

## Experiment One

### Materials and Methods

#### Design

A within-subjects design with the factors 2 Condition (natural history, placebo) × 2 Test (pre-test; T1, post-test; T3) × 2 Order of conditions (PC first or NHC first) × 2 Participant sex × 3 Experimenter sex (female, male, and the both male and female) was used. The first two factors were within-subjects’ factors, and the last three factors were between-subjects factors. Participant sex and Experimenter sex were entered as independent variables in the analyses.

#### Participants

Participants were recruited from the University of Tromsø (*N* = 71) through flyers and advertisements. Potential participants sent an email to the first author and after checking the eligibility based on the inclusion criteria, were enrolled to the study. Pregnancy, somatic and psychiatric disorders, and use of prescription-based or allergy medications, except for birth control pills, led to the exclusion from the experiment. All participants were asked to abstain from nicotine or caffeine 3 h before the experiment. Of 71 participants, 12 were excluded due to reporting pain intensity on a numerical rating scale (NRS) below “one” (outlier cases) in T1s. Thus, 59 participants (26 females, age range = 19–27 years [*M_*age*_* = 21.46, *SD* = 2.17]; 33 males, age range = 19–34 years [*M*_*age*_ = 24.21, *SD* = 4.02]) were included in the analyses. All participants received a gift certificate of 300 Norwegian Kroner (about 50 USD).

#### Outcome Variables

##### Subjective reports of pain intensity and unpleasantness

In both experiments, subjective pain intensity and unpleasantness reports were recorded on an 11-digit NRS, where for pain intensity, ‘0’ was anchored as “no pain,” and ‘10’ as “the most intense pain imaginable.” For pain unpleasantness, ‘0’ was anchored as “no unpleasantness” and ‘10’ as “unbearable unpleasantness.” The difference between pain intensity and pain unpleasantness was explained according to Price et al. (1983). Participants were told: “the pain intensity is how strong the pain feels, but the pain unpleasantness is how disturbing the pain is for you.” Think of listening to a sound, such as a radio. As the volume of the sound increases, I can ask you how loud it sounds or how unpleasant it is to hear it. The intensity of pain is like loudness; the unpleasantness of pain depends not only on intensity but also on other factors which may affect you (Price et al., 1983). Participants were also asked to judge the two aspects independently.

##### Subjective reports of stress and arousal

In both experiments subjective stress and arousal were measured using the Norwegian translation of The Short Adjective Check List (SACL; Mackay et al., 1978). The two adjective pairs for stress (tense-relaxed and nervous-calm) and two for arousal (sleepy–awake and tired-energetic) from SACL are shown to be reliable and timely scales for stress and arousal (O’Neill and Parrott, 1992; Parrott, 1995). Moreover, the conversion of SACL stress and arousal pairs to numeric rating versions have been shown to be faster in administration and to be good alternatives for longer inventories (Lang, 1980; Wade et al., 1990). The numeric rating version of these two pairs have been also successfully used to measure subjective stress and arousal in previous similar studies (e.g., Aslaksen and Flaten, 2008). The adjective pairs were reported on an 11-digit NRS where ‘0’ for stress scale indicated “completely relaxed/calm” and for arousal indicated “completely sleepy/tired.” On the other end, ‘10’ for the stress scale indicated “maximally tense/nervous” and for the arousal indicated “maximally awake/energetic.” The mean score of the sum of the two adjective pairs for each scale were used as the subjective stress and arousal ratings.

##### Blood pressure

An automatic blood pressure (BP) monitor (BP A100 Plus, Microlife, Switzerland; Bonso et al., 2009) was used to record systolic blood pressure (SBP), and diastolic blood pressure (DBP) (Berntson et al., 2017) in both experiments. Only one reading was taken in each recording. The experimenter performed the measurements, was present in the room during the BP readings and the participants could see the reading during the measurement.

#### Procedure and Interventions

Both experiments were conducted inside a steel cubicle (2.8 × 2.8 m), with a constant temperature of 20°C. All participants were tested between 8 AM and 5 PM, while seated in a comfortable chair. The verbal communication between experimenters and participants in both experiments were standardized using a written protocol. All participants in both experiments were informed that they could terminate the experiment at any time and for whatever reason by pushing a button within reach.

A repeated measures design was used in *Experiment One*. Participants underwent both a “placebo condition (PC)” and a “natural history condition (NHC)” in a randomized order and on the same day with a 5-min interval between the conditions. The placebo condition was run first for 29 participants, and second for the remaining 30 participants. Each condition consisted of three phases: a pre-test (T1), a manipulation trial (in the PC; T2) or control (in the NHC; T2), and a post-test (T3). There was a 5-min break between each phase. Baseline temperature was 32°C, and painful stimulation was applied at 54°C for about 0.1 s in T1 and T3, and at 51°C for about 0.1 s in T2. The temperature rise rate was 70°C/s and the cooling rate was 40°C/s. Participants were administered 15 painful stimulations with a total duration of 3 min. The interstimulus interval (ISI) was randomly varied between five and 15 s. The location of the thermode was moved in a pre-defined pattern after each stimulation was administered.

**FIGURE 1:**
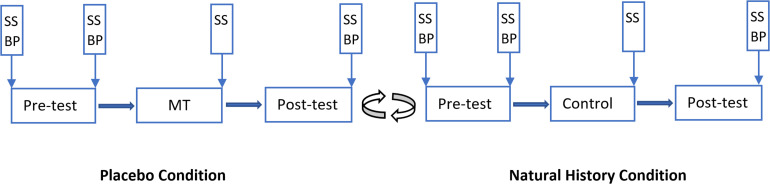
Overview of the *Experiment One.* Vertical boxes: spots where subjective stress/arousal (SS) and/or blood pressure (BP) were measured. There was a 5-min break between phases and between conditions. MT, manipulation trial. The placebo condition was presented to 29 participants as the first condition, and to 30 participants as the second condition.

Participants were told that “this study is investigating the effects of a pain-relieving cream on heat pain, so in some points in the experiment, you will receive either a pain-relieving cream or an inactive cream before the pain is administered.” Participants were also informed how to report pain and fill in other scales. In T1, participants received thermal pain to volar forearm for 3 min. Before T2 in the PC, a placebo cream was administered with the information that “the cream is a potent painkiller with excellent effects on short dermal heat pain.” After application of the cream, the painful stimulation was surreptitiously lowered compared to T1, to 51°C, to associate the cream with lower pain perception. The placebo cream with the same information was also applied before T3 in PC but with the same pain stimulation of 54°C as in T1. The procedure for NHC was identical to PC except that the temperature was kept at 54°C, and that no cream and no information was provided to the participants during the phases. Subjective ratings of pain intensity and pain unpleasantness were obtained after each stimulation. Subjective stress/arousal were recorded before T1 and after T1, T2 and T3. BP was recorded before T1 (baseline), and after T1 and T3 (Figure 1). Like previous studies (Aslaksen et al., 2014, 2015), only one reading was taken in each measurement point. The experimenter(s) performed the measurements and were present in the room at the time of measurement. To test the effects of the Participant and Experimenter sex as independent factors, three testing blocks were arranged based on the sex of participants and experimenters. Thus, of 59 included participants, 15 participants (six *male* and nine *female* participants) were tested by a female experimenter; and 16 participants (eight *male* and eight *female* participants) were tested by a male experimenter. For the other 28 participants (19 *male* and 9 *female* participants) a male and a female experimenter were both present in the testing room throughout the experiment.

#### Apparatus

A CHEPS (Medoc Ltd, Ramat Yishai, Israel) with a 27 mm diameter thermofoil thermode induced thermal pain to the right forearm of participants.

#### Experimenters

Four experimenters conducted the study (two *females*) (age Range 23–25 [*M* = 24.25, *SD* = 0.96]). The experimenters were trained with the experiment equipment and the procedure, but did not get any information about the research questions or hypotheses of the study. The experimenters were instructed to not to give any further information about the study than what was stated in the consent form. The experimenters in both studies did not know whether the participants received active or inactive medication and the study was run double-blind.

#### Ethics

Both studies were approved by the Regional Committee for Medical Research Ethics North Norway (*Experiment One* Project Nr. 2010/3309; *Experiment Two* Project Nr: 2012/1888) and were run at the Department of Psychology at the University of Tromsø. All participants provided a signed written informed consent.

#### Placebo Definition

The main outcomes in both experiments were pain intensity and pain unpleasantness. The placebo effects for pain intensity and unpleasantness were calculated as the difference between the pain intensity reported in the T1 and the T3 in the placebo condition/group compared to the natural history condition/group(s). Placebo effects on stress, arousal and BP were defined the same way.

#### Randomization

The Random Number Generator (RNG) generated numbers from 1 to 6 for group assignment and participant sex, and these numbers were used to define group assignment and to assign an equal number of males and females to each group in both experiments. For *Experiment One* the number ‘1’ indicated female in the group 1, the number ‘2’ indicated male in the group 1, the number ‘3’ indicated male in the group 2, and number ‘4’ indicated female in the group 2. After (relatively) equal numbers of males and female participants were assigned to two groups, RNG was used to specify the order of conditions (i.e., PC or NHC as the first condition). So, number 1 indicated that group 1 had to undergo the PC first and group 2 the NHC first; and number 2 indicated that the group 2 had to undergo the PC first and group 1 the NHC first.

To assign a (relatively) balanced number of male and female participants for *Experiment Two’s* groups, the number ‘1’ indicated female in the placebo group and the number ‘2’ indicated male in the Placebo group. Following the same order, the numbers ‘3’ and ‘4’ and the numbers ‘5’ and ‘6’ were used for the assignment of females and males to the Cream-control and Pain-control groups. This procedure was repeated until all the participants were assigned to group/condition in both experiments. This was done to ascertain that the same group and sex were not tested more than two times in a row.

#### Blinding

Both experiments were run double-blind, so neither the participants nor the experimenters knew whether active medication or placebo was administered. In *Experiment One* three participants, and in *Experiment Two* four participants received an active pain-relieving cream (Emla cream, 2.5 g) to assure blinding. The seven participants who received Emla were then excluded from the analyses.

#### Statistical Power

A previous between-subjects study (Aslaksen et al., 2011) testing the effects of placebo manipulations on pain unpleasantness, reported an effect size of 0.478 for placebo effects (Natural history group *M* = 3.42, *SD* = 1.52, Placebo group *M* = 2.73, *SD* = 1.37). Assuming a statistical power (1 – beta) of 0.8, alpha level set as 0.05 and with an expected effect size of 0.478 for an ANOVA repeated measures within-between factors design, a total sample size of 24 was required for *Experiment One*. However, considering the possibility of outliers and withdrawal, a larger sample of 59 participants were recruited. Regarding *Experiment Two*, using the same parameters for an ANOVA between-within factors design, a total sample size of 84 was required. However, considering the same rationale as in *Experiment One*, a larger sample of 98 participants were recruited.

#### Statistical Analyses

In both experiments all analyses were performed using IBM SPSS Statistics 27 (SPSS, Inc, Chicago, IL, United States). Data were analyzed using repeated measures analysis of variance (ANOVA). The method suggested by Hoaglin and Iglewicz (1987) was used to detect outlier data, and the detected outliers were resolved using Winsorising technique (Dixon, 1980). For *Experiment One*, the variables Condition with two levels (PC and NHC) and Test with two levels (T1 and T3) were entered as within-subjects variables. Since lower temperatures were administered in T2, data for T2 was not entered in the analyses. Participant sex with two levels and Experimenter sex with three levels were entered as between-subjects’ factors.

For *Experiment Two*, the variables Group with three levels, Participant and Experimenter sex each with two levels were entered as between-subjects factors, and the variables Stimulus intensity (intensity levels 2, 4, and 6) with three levels and Test with three levels (T1, T2 and T3) were entered as within-subjects factors. In *Experiment Two* analyses, T2 was included, since participants in PG and PCG received identical pain stimulations in T2 and that the pain stimulations in CCG T2 was not lowered, as they were lowered in PG and PCG T2s. The data for CCG T2 was compared with the data for T2 PG and T2 PCG in separate contrast analyses (for more info please see *Experiment Two* Methods). Then, and for both experiments, all significant interactions were followed up with contrast analyses employing Fisher’s LSD statistics.

To test whether conditioning took place in the placebo condition/groups, the correlation between the difference of the pain reports in T1 and T2, i.e., the unconditioned response (UR), and the conditioned response (CR), i.e., the difference between the pain report in T1 and T3, i.e., the placebo effect, was investigated. The UR was defined as the difference in the pain report between T1 and T2, as the conditioned stimuli (CS; the cream) signals this difference (or reduction) in pain. Previous research has found a correlation between CR amplitude and UR amplitude, so a larger UR gives rise to a larger CR (e.g., Passey, 1948; Solomon et al., 1986; Reber et al., 1991; Grillon and Hill, 2003).

Moreover, mediation analyses following the recommendations of Baron and Kenny (1986) were used to investigate the effects of expectations and stress in *Experiment Two*.

### Results

Means for pain intensity, unpleasantness, stress, arousal, systolic and diastolic blood pressure data across conditions and phases are provided in Table 1.

**TABLE 1:** Pain intensity, unpleasantness, stress, arousal, systolic and diastolic blood pressure data across conditions and phases.

Condition	Phase	PI	PU	SS	SA	SBP	DBP
Placebo (M; SE)	Baseline	–	–	2.85; 0.18	5.45; 0.21	125.08; 1.78	78.07; 0.95
	Pre-test (T1)	3.17; 1.64	2.98; 1.79	3.03; 1.47	5.38; 1.78	123.32; 2.08	76.44; 0.96
	MT (T2)	1.78; 1.30	1.57; 1.20	2.43; 1.40	4.94; 1.76	–	–
	Post-test (T3)	3.14; 1.80	2.92; 1.80	2.59; 1.46	4.91; 1.73	122.86; 1.62	77.22; 1.01
Natural history (M; SE)	Baseline	–	–	2.86; 0.21	5.27; 0.21	128.19; 1.85	77.97; 8.86
	Pre-test (T1)	3.00; 1.47	2.71; 1.44	3.02; 1.46	5.45; 1.72	124.62; 1.54	78.17; 8.51
	Control (T2)	3.02; 1.65	2.75; 1.66	2.75; 1.37	5.19; 1.71	–	–
	Post-test (T3)	2.97; 1.63	2.78; 1.64	2.62; 1.50	5.16; 1.70	126.02; 1.71	81.14; 17.09

*M, mean; SE, standard error; MT, manipulation trial; PI, pain intensity; PU, pain unpleasantness; SS, subjective stress; SA, subjective arousal; SBP, systolic blood pressure; DBP, diastolic blood pressure. BP was recorded before and after Pre-test (T1), and after Post-test (T3).*

#### Pain Unpleasantness and Intensity

##### Pain unpleasantness

A significant interaction of Participant sex and Condition [*F*(1,52) = 7.28, *p* = 0.007, $\eta_{\text{p}}^{2}$ = 0.10] showed that female participants had higher pain unpleasantness reports in the PC compared to the NHC (*Mean diff* = –0.12, *SE* = 0.16, *p* = 0.002). No other main or interaction effect were significant.

##### Pain intensity

A similar significant interaction of Participant sex and Condition [*F*(1,51) = 5.82, *p* = 0.02, $\eta_{\text{p}}^{2}$ = 0.10] showed that female participants had higher pain intensity reports in the PC compared to the NHC (*Mean diff* = –0.49, *SE* = 0.17, *p* = 0.006). As the Order of conditions was not significant as a main effect, and did not interact with the other factors, it was excluded from the analyses. No other main or interaction effect were significant.

No placebo effect was observed on pain. Thus, the first and second hypotheses were not supported. Next, to see whether conditioning took place in the PC, the correlation of the UR and CR amplitudes were investigated.

In the PC, the pain intensity UR correlated with the pain intensity CR [*r*(57) = 0.53, *p* = 0.0001]. However, in the NHC there was also a correlation between the difference of the pain reports in the T1 and T2, and the difference between the pain report in T1 and T3 [*r*(57) = 0.53, *p* = 0.0001].

#### Subjective Stress

Subjective stress decreased over Tests [*F*(1,53) = 17.69, *p* = 0.0001, $\eta_{\text{p}}^{2}$ = 0.26] with no difference between the conditions. No other theoretically interesting main effects or interactions were found in subjective stress data.

#### Subjective Arousal

Arousal decreased over Tests [*F*(1,53) = 11.61, *p* = 0.001, $\eta_{\text{p}}^{2}$ = 0.18] with no difference between the conditions.

#### Systolic Blood Pressure

Females had lower SBP than male participants [*F*(1,51) = 9.29, *p* = 0.004, $\eta_{\text{p}}^{2}$ = 0.15]. Also, the significant main effect of Condition [*F*(1,51) = 8.28, *p* = 0.006, $\eta_{\text{p}}^{2}$ = 0.14] showed that participants had lower SBP in the PC compared to the NHC (*Mean diff* = 3.43, *SE* = 1.19, *p* = 0.006).

#### Diastolic Blood Pressure

**FIGURE 2:**
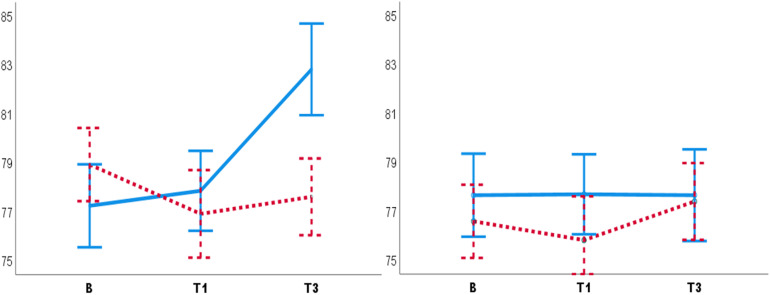
Diastolic blood pressure for males **(left panel)** and females **(right panel)**. Male participants had lower DBP in the PC T3 compared to the NHC T3 **(left panel)**. No such placebo response was observed for females **(right panel)**. B, baseline. T1, pre-test. T3, post-test. Blue lines: NHC (*N* = 30). Red dashed lines: PC (*N* = 29). Error bars: ±1 *SE*.

**FIGURE 3:**
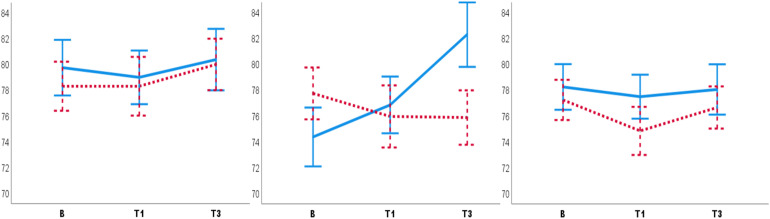
Diastolic blood pressure recorded by the male **(left panel)**, the female **(middle panel)** and both male and female experimenters **(right panel)**. Female experimenters **(middle panel)** recorded lower DBP in PC T3 as compared to NHC T3. B, baseline. T1, pre-test. T3, post-test. Blue lines: NHC. Red dashed lines: PC. Error bars: ±1 *SE*.

The significant interaction of Condition, Test and Participant sex [*F*(2,51) = 4.02, *p* = 0.02, $\eta_{\text{p}}^{2}$ = 0.13] showed that male participants had lower DBP in the PC T3 compared to the NHC T3 (*Mean diff* = 5.19, *SE* = 1.69, *p* = 0.003) (Figure 2). The significant interaction of Condition, Test, and Experimenter sex [*F*(4,104) = 3.78, *p* = 0.03, $\eta_{\text{p}}^{2}$ = 0.11] showed that the female experimenter recorded lower DBP in the PC T3 compared to the NHC T3 (*Mean diff* = 6.41, *SE* = 2.27, *p* = 0.007) (Figure 3). No other main effects or interactions were significant in the BP data.

### Discussion

*Experiment One* failed to support our first and second hypotheses as it fell short to generate a placebo effect on pain reports. Therefore, the effects of participant and experimenter sex on placebo effects on pain report remained unanswered. Next, we discuss possible reasons for this result and other findings from *Experiment One*.

There was no placebo response in the pain data. Surprisingly, pain intensity and unpleasantness were higher in females in the PC compared to NHC, but this was true for both the T1 and T3. However, there were placebo effects in physiological stress, as male participants had lower DBP following the placebo manipulation and, the female experimenter recorded lower DBP in the PC T3 compared to NHC T3.

#### Pain Reports

The females’ higher pain in the PC than the NHC is hard to explain as the study was run within-subjects. Thus, no pre-existing group differences can explain this finding. There was also no evidence of habituation to painful stimulation.

*Experiment One* was modeled after a similar repeated-measure study that successfully observed a placebo response (Aslaksen and Flaten, 2008). In that study, the two conditions were run on different days, which is different from the present study where both conditions were run on the same day. Therefore, we assume that the failure to generate a placebo effect, and probably the higher pain in the PC than the NHC reported by females are due to the methodological and procedural details of the study, e.g., due to the repeated measures design. We have at present no better explanation for the higher pain report in females in PC in the present study. Surprisingly, the participant and experimenter sex affected BP data, suggesting a possible placebo response taking place on physiological stress.

#### Physiological and Subjective Stress

##### Participant sex

Male participants had lower DBP in the PC T3 compared to the NHC T3, suggesting a placebo response on DBP in males only. This finding is against the third hypothesis, that following the placebo manipulations females would have lower stress levels. A review by Vambheim and Flaten (2017) showed that males are more responsive to verbal placebo manipulations on pain, whereas Enck and Klosterhalfen (2019) suggested that females are more responsive to classical conditioning. None of these hypotheses were supported. A finding of a placebo effect on DBP is not common, given that investigators often only analyze SBP in pain and placebo studies, due to the assumption that DBP is highly affected by respiration, and would not give valuable information (e.g., Sved, 2009) (for a review see Daniali and Flaten, 2020). On the contrary, the present finding can be evidence suggesting DBP as an index in detection of placebo responses on physiological stress. Female participants had lower SBP than male participants, which is reported previously (e.g., Aslaksen et al., 2014, 2015).

##### Experimenter sex

In the presence of female experimenters, lower DBP was observed in T3 in the PC compared to T3 in the NHC, i.e., a placebo response. This finding confirms our fourth hypothesis that lower stress is recorded by female experimenters. Previous research has reported that being tested by female experimenters/clinicians can lower pain reports (e.g., Fillingim et al., 1999; Carter et al., 2002). The present finding suggests that being tested by a female experimenter can also lead to lower physiological stress (as indexed by DBP). However, this finding is not conclusive, as only one male and female experimenter was used in each condition.

#### Limitations and Conclusion

The results from *Experiment One* showed no placebo effects on pain reports, so, the first and second hypotheses on the effects of participant and experimenter sex on placebo effects on pain reports were not supported. However, *Experiment One* showed findings on the effects of participant and experimenter sex on DBP, that were suggestive of a placebo response on physiological stress. The lower DBP in males in PC contradicted the third hypothesis. The results were also suggestive of a distinction between placebo effects on pain and on stress associated with pain, which was not hypothesized originally. However, the present findings are associated with uncertainty, as the observation of such results could be due to other factors, such as the within-subjects design of the study, using only one experimenter from each sex, and measuring BP only one time in each measurement point, and low statistical power.

## Experiment Two

As our hypotheses were not supported in *Experiment One*, another experiment was carried out to test the hypotheses and to see if the results from *Experiment One* could be replicated. To avoid the previous shortcomings, we employed a mixed-design with two experimenters from each sex, to further investigate the effects of participant and experimenter sex in placebo effects on pain and on stress. Identical to *Experiment One*, the main outcomes were pain intensity and pain unpleasantness, and the secondary outcomes were subjective reports of stress and arousal, and BP as the physiological stress index. Moreover, the expected effectiveness of the placebo cream was also measured in *Experiment Two*. Pain was induced by brief electrical stimuli, and the intensity of the stimuli were varied to investigate if placebo effects could be observed at different levels of pain.

### Materials and Methods

#### Design

A 3 Group (Placebo, PG; Pain-control, PCG; Cream-control, CCG) × 2 Participant’s sex × 2 Experimenter’s sex × 3 Stimulus intensities (2, 4, 6) × 3 Tests (pre-test, T1; manipulation trial/control, T2; post-test, T3) mixed-design was used. The first three factors were between-group factors, the last two factors were within-group factors.

#### Participants

Participants (*N* = 98), between the age of 18 and 40 years were recruited from University of Tromsø, using the same recruitment method and same inclusion and exclusion criteria as in *Experiment One*. Five participants were excluded due to reporting pain intensity lower than “one” in the T1. Thus, 93 participants (50 females, 43 males) were included in the analyses. Unfortunately, due to an error in storage of individual’s demographic information, data related to age was lost. However, all participants were between the age of 18 and 40, as being under/over that age led to exclusion, based on documents showing that the increase in age might affect pain sensitivity (e.g., Yezierski, 2012). Participants received a gift certificate of 200 Kroner (approximately 35 US dollars).

#### Outcome Variables

##### Subjective reports of pain intensity, unpleasantness, stress, and arousal

The same subjective measurements as in *Experiment One* were used in *Experiment Two*.

##### Cream effectiveness

Cream effectiveness was measure by asking: “On a scale from ‘0’ to ‘10,’ where ‘0’ is no reduction in pain, and ‘10’ is maximum reduction in pain, how much do you expect the cream to reduce your pain?”

##### Blood pressure

Blood pressure was tested with the same system as in *Experiment One*. Only one reading was taken in each recording. The experimenter performed the measurements, was present in the room while taking BP readings and the participants could see the reading during the measurement.

#### Procedure

During a calibration procedure, the pain threshold and four different levels of pain intensity (levels 1, 2, 4, and 6, based on an NRS) were calculated individually. Pain threshold was calculated by starting with administration of a stimulus intensity of 0.03 mA and gradually increasing the intensity until the participant reported pain intensity equal to ‘1’ on the NRS. The stimulus intensity thereafter increased gradually until the participant reported pain intensity equal to 2, 4, and 6 on the NRS. The procedure was repeated once more, but in a descending order, starting with the mA-strength representing level 6, and ending with the mA-intensity of ‘1.’ The mean mA-intensity of the two calibration procedures (ascending and descending) were calculated and used in the experiment. Hence, the mean of the two mA-intensities equaling 2, 4, and 6 were used in the experiment.

After pain intensity was calibrated, participants were randomly assigned to a Placebo group (PG; *N* = 32; 15 *males*, 17 *females*), a Cream-Control group (CCG; *N* = 31; 14 *males*, 17 *females*), or a Pain-Control group (PCG; *N* = 30; 14 *males*, 16 *females*), that each included a pre-test (T1), a manipulation trial (in the PG) or a control (in the CCG and PCG) (T2), and a post-test (T3).

Participants were verbally informed that “depending on the group you are assigned to, you may or may not receive the cream containing a medication (named ‘*Embla*’) with a powerful and quick relieving effect.” The cream administered to the PG and the CCG was in fact an inactive cream filled in tubes identical to Emla cream by the hospital pharmacy at the University hospital of North Norway (UNN). All groups received identical treatment except in the manipulation trial/control phase (T2). In T1 and T3 in all groups, 18 stimulations of three different intensities (2, 4, and 6) were administered. The intensity of the stimulations varied due to an identical pre-defined pattern within the tests to enable comparison of pain levels in the T1 and T3. The interstimulus interval varied between eight to 12 s. After the T1, an envelope with information about group assignment was opened in the presence of the participants, to make sure that participants were given identical treatment during T1. In the T2s, four stimulations of three different intensities, 1, 2, 4 intensity stimuli for the PG and PCG, and 2, 4, and 6 intensity stimuli for the CCG, were administered.

Participants in the PG and the CCG received the placebo cream. After application of the cream in the PG T2, the painful stimulation was surreptitiously lowered from 2, 4, and 6 to 1, 2, and 4, respectively, to associate the cream with lower pain experiences. The same cream and information were re-administered in the PG T3, but this time the stimulus intensity levels corresponding to 2, 4, and 6 were administered. The PG acted as the experimental group and the other two were control groups. The CCG controlled for effects of the application of the cream, by applying the cream as in the PG, but holding the intensity at the same levels in all three tests. The PCG controlled for the lower intensities in T2, by reducing the intensities in T2 as in the PG, but without the application of the cream.

**FIGURE 4:**
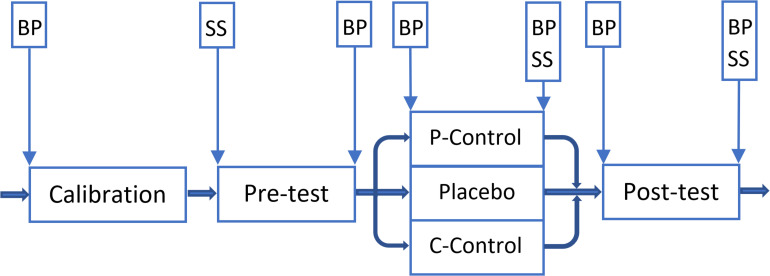
Overview of the *Experiment Two.* Vertical boxes: spots where subjective stress/arousal (SS) and/or blood pressure (BP) were measured. A 5-min break was given to participants between phases. P-Control: PCG. Placebo: PG. C-Control: CCG.

After application of the cream in both the T2 and T3 of PG and CCG, participants rated how much they expected the cream to reduce the pain on an NRS. After each pain stimulation, participants rated how intense and unpleasant each pain stimulus was using an NRS. Subjective stress and arousal were measured before the T1 (baseline), and after T2 and T3. BP was recorded before the calibration procedure (baseline), after the T1, and before and after the T2 and T3 (total of six measurements) (Figure 4). The expected effectiveness of the cream was measured before T2 and before T3. The experimenters tested 19 participants each (8 or 9 females each). Female experimenters tested 48 (22 *male* and 26 *females*); and male experimenters tested 45 (21 *males* and 24 *females*) participants.

#### Apparatus

A Digitimer stimulator (DS7A) induced electrical stimulations via an electrode to the right forearm. The electrode originally designed by Mouraux et al. (2010) was produced by engineers at the department and used in the present study.

#### Experimenters

Five experimenters (two *females*) between the age of 24 and 33 (*M*_*age*_ = 30.20; *SD* = 1.8) conducted the study (for more info see section ‘Experimenters’ in *Experiment One*).

Information regarding the Ethics, Placebo definition, Blinding, Randomization and the Statistical power and statistical analyses are provided in the *Experiment One*’s “*Materials and Methods.*”

### Results

Means and standard errors (SDs) for pain intensity and unpleasantness reports for the three stimulus intensities and means and SDs for subjective and physiological stress are shown in Table 2.

**TABLE 2:** Pain intensity, unpleasantness, stress, arousal, systolic, and diastolic blood pressure data across groups and phases.

Group	Phase	PI	PU	SS	SA	SBP	DBP
	Calibration	–	–	–	–	127.65; 2.30	76.65; 1.80
Placebo (M; SE)	Pre-test (T1)	(2) 2.64; 1.17 (4) 3.73; 1.18 (6) 4.61; 1.08	(2) 1.80; 1.25 (4) 2.61; 1.36 (6) 3.45; 1.52	2.26; 1.71	5.78; 1.69	124.47; 1.77	76.78; 1.82
	MT (T2)	(1) 1.20; 0.99 (2) 1.81; 0.95 (4) 3.03; 2.10	(1) 0.60; 0.65 (2) 0.95; 0.79 (4) 3.03; 2.10	1.45; 1.39	5.17; 1.98	(1) 124.24; 2.15 (2) 124.83; 1.74	73.83; 1.53 75.21; 1.47
	Post-test (T3)	(2) 2.11; 1.08 (4) 3.23; 1.14 (6) 4.43; 1.26	(2) 1.28; 0.97 (4) 2.16; 1.28 (6) 3.26; 1.81	1.30; 1.37	4.96; 1.70	(1) 125.76; 2.00 (2) 123.90; 2.01	73.97; 1.95 73.93; 1.80
	Calibration	–	–	–	–	125.35; 2.27	74.53; 1.38
Pain-control (M; SE)	Pre-test (T1)	(2) 2.45; 1.21 (4) 3.68; 1.70 (6) 4.59; 1.86	(2) 1.87; 1.16 (4) 3.00; 1.73 (6) 3.77; 1.85	2.58; 1.52	5.64; 1.63	122.77; 1.69	76.10; 1.12
	MT (T2)	(2) 1.60; 1.09 (4) 2.25; 1.16 (6) 3.51; 1.34	(2) 1.25; 0.94 (4) 1.66; 1.14 (6) 3.51; 1.34	2.01; 1.32	5.07; 1.87	(1) 122.60; 1.61 (2) 120.97; 1.93	74.33; 1.19 74.03; 1.37
	Post-test (T3)	(2) 2.12; 1.30 (4) 3.33; 1.44 (6) 4.45; 1.73	(2) 1.72; 1.27 (4) 2.66; 1.48 (6) 3.86; 1.64	1.93; 1.36	4.87; 1.74	(1) 120.03; 1.56 (2) 123.53; 1.85	74.20; 1.44 75.70; 1.33
	Calibration	–	–	–	–	128.10; 2.14	77.90; 1.54
Cream-control (M; SE)	Pre-test (T1)	(2) 2.43; 1.58 (4) 3.46; 1.59 (6) 4.35; 1.47	(2) 1.90; 1.68 (4) 2.64; 1.71 (6) 3.43; 1.67	2.27; 1.17	5.27; 1.66	123.68; 1.84	75.87; 1.38
	MT (T2)	(1) 1.64; 0.96 (2) 2.69; 1.38 (4) 3.51; 1.51	(1) 1.11; 1.04 (2) 1.89; 1.29 (4) 3.51; 1.51	2.00; 1.48	4.67; 1.67	(1) 123.00; 1.67 (2) 124.00; 1.89	74.39; 1.38 77.06; 1.60
	Post-test (T3)	(2) 2.05; 1.34 (4) 3.03; 1.38 (6) 4.13; 1.57	(2) 1.53; 1.54 (4) 2.31; 1.58 (6) 3.31; 1.87	1.56; 1.13	4.54; 1.78	(1) 125.84; 4.37 (2) 123.79; 1.98	77.90; 2.56 77.76; 1.84

*M, mean; SE, standard error; MT, manipulation trial; PI, pain intensity; PU, pain unpleasantness; SS, subjective stress; SA, subjective arousal; SBP, systolic blood pressure; DBP, diastolic blood pressure. Subjective stress and arousal were tested before pre-test, and after manipulation trials and post-tests. Systolic and diastolic BP were tested before calibration, after pre-test, and before (1) and after (2) manipulation trials and post-tests.*

#### Pain Unpleasantness and Intensity

##### Pain unpleasantness

Pain unpleasantness decreased over Tests [*F*(1,166) = 23.15, *p* = 0.0001, $\eta_{\text{p}}^{2}$ = 0.21] with no difference between Groups. The significant interaction of Experimenter sex and Group [*F*(2,83) = 5.18, *p* = 0.008, $\eta_{\text{p}}^{2}$ = 0.11] showed that female experimenters received lower pain unpleasantness in the PG (*Mean diff* = –1.49, *SE* = 0.40, *p* = 0.000) and in the CCG (*Mean diff* = –0.91, *SE* = 0.45, *p* = 0.04) compared to the PCG. No other main effects or interactions were significant. The main effect of Stimulus intensity was significant [*F*(2,82) = 190.36, *p* = 0.0001, $\eta_{\text{p}}^{2}$ = 0.82]. There were no significant differences between groups and stimulus intensities.

##### Pain intensity

Pain intensity decreased over Tests [*F*(1,166) = 53.96, *p* = 0.0001, $\eta_{\text{p}}^{2}$ = 0.39] with no difference between Groups. The significant interaction of Experimenter sex and Group [*F*(2,83) = 3.49, *p* = 0.03, $\eta_{\text{p}}^{2}$ = 0.07] showed that the presence of female experimenters led to lower pain reports in the PG (*Mean diff* = –1.00, *SE* = 0.37, *p* = 0.008) and in the CCG (*Mean diff* = –0.85, *SE* = 0.41, *p* = 0.04) compared to the PCG. No other main effects or interactions were significant.

The main effect of Stimulus intensity was significant [*F*(2,82) = 156.57, *p* = 0.0001, $\eta_{\text{p}}^{2}$ = 0.79]. There were no significant differences between groups and stimulus intensities in T1 and T3s. Moreover, the interaction of Group and stimulus intensity levels in T2 was not significant.

Interestingly, based on the multivariate ANOVA results, there was no difference between CCG T2 and PCG T2, even though lower pain stimuli were induced in PCG compared to CCG. This insignificant difference between CCG and PCG in T2s was present for all three stimuli intensities (intensity level 2: *Mean diff* = –0.90, *SE* = 0.27, *p* = 0.74; intensity level 4: *Mean diff* = 0.25, *SE* = 0.30, *p* = 0.41; and, intensity level 6: *Mean diff* = 0.23, *SE* = 0.44, *p* = 0.60).

Thus, our first and second hypotheses that females should display higher placebo effects than males, and that higher placebo effects are related to being tested by the opposite sex, were not supported.

There were significant UR-CR correlations in the PG [e.g., for medium-intensity = *r*(32) = 0.52, *p* = 0.01] and the PCG [e.g., for high-intensity = *r*(31) = 0.40, *p* = 0.02]; and in the CCG, the correlations between the difference between T1 and T2, and the difference between T1 and T3 were significant [e.g., for high-intensity = *r*(32) = 0.48, *p* = 0.005].

##### Expectancy of cream effectiveness

The main effect of Test showed that expectancy of cream effectiveness decreased from T2 to T3 [*F*(1,52) = 9.72, *p* = 0.003, $\eta_{\text{p}}^{2}$ = 0.15]. The significant interaction of Test by Group [*F*(1,51) = 4.87, *p* = 0.03, $\eta_{\text{p}}^{2}$ = 0.09] showed that in the PG, participants expected lower cream effectiveness in T3 compared to T2 (*Mean diff* = 2.06, *SE* = 0.45, *p* = 0.002). No differences in cream effectiveness between the PG and the CCG were observed (Table 3). The main effect of Experimenter sex [*F*(1,52) = 4.06, *p* = 0.049, $\eta_{\text{p}}^{2}$ = 0.07] showed that female experimenters received higher expectations of cream effectiveness compared to male experimenters (*Mean diff* = 1.33, *SE* = 0.65, *p* = 0.049).

**TABLE 3:** Cream effectiveness in the Placebo group (PG) and Cream-control group (CCG).

Group	Phase	Cream effectiveness
Placebo (M; SE)	MT (T2)	3.41; 0.62
	Post-test (T3)	1.26; 0.37
Cream-control (M; SE)	MT (T2)	1.65; 0.39
	Post-test (T3)	1.29; 0.32

*M, mean; SE, standard error; MT, manipulation trial.*

##### Mediation effects of expectancy

In the presumed mediation model, the pain intensity UR was entered as the predictor and the pain intensity CR (the placebo effect) as the outcome variable. The expected cream effectiveness reported in T3 was used as a mediator between the pain intensity UR and the pain intensity CR. The model was then tested for the three stimulus intensities in the PG. As the participants in the CCG received the cream, the mediating effects of expected cream effectiveness on the effects of the pain intensity UR on the CR was tested using a similar model as the one used for the PG. Overall, the results showed that at none of the stimulus intensity levels in the PG or the CCG, the expected cream effectiveness acted as a significant mediator for the relationship between the pain intensity UR and pain intensity CR.

Since the pain report in the CCG T2 was not different from the PCG T2, the difference between the pain report in T1 and T2 was regressed on the expected cream effectiveness reported in T2. For all three stimulus intensity levels, the expected cream effectiveness data predicted the lowered pain in the CCG T2 (Intensity level 2: *B* = 0.23, *SE* = 0.09, β = 0.41, *p* = 0.02, *R*^2^ = 0.14; intensity level 4: *B* = 0.30, *SE* = 0.09, β = 0.51, *p* = 0.003, *R*^2^ = 0.23; intensity level 6: *B* = 0.48, *SE* = 0.09, β = 0.68, *p* = 0.0001, *R*^2^ = 0.44).

#### Subjective Stress

**FIGURE 5:**
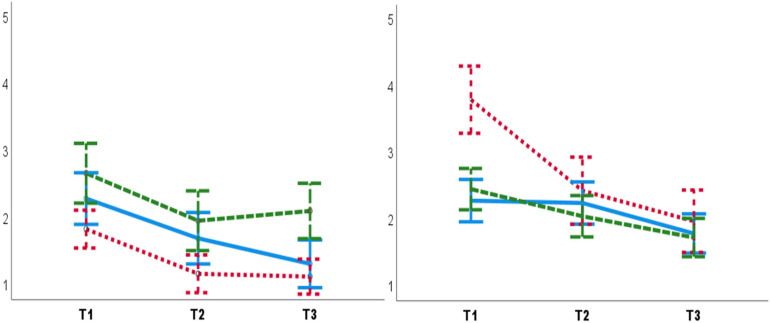
Lower subjective stress was reported to female experimenters **(left panel)** in the PG T3 (post-test) compared to the female experimenters in the PCG T3. Higher subjective stress was reported to male experimenters **(right panel)** in the PG T1 (pre-test), compared to male experimenters in T1. T1: pre-test. T2: manipulation trial/control. T3: post-test. Blue lines: CCG. Green dashed lines: PCG. Red dashed lines: PG. Error bars: ± 1 *SE*.

Subjective stress decreased over Tests [*F*(2,164) = 55.26, *p* = 0.0001, $\eta_{\text{p}}^{2}$ = 0.40]. The significant interaction of Experimenter sex, Group, and Tests [*F*(4,164) = 4.14, *p* = 0.004, $\eta_{\text{p}}^{2}$ = 0.09] showed that lower subjective stress were reported to female experimenters in the PG T3 compared to the female experimenters in the PCG T3 (*Mean diff* = –0.97, *SE* = 0.48, *p* = 0.04) (Figure 5). Moreover, higher subjective stress was reported to male experimenters in the PG T1 compared to male experimenters in the CCG (*Mean diff* = 1.51, *SE* = 0.59, *p* = 0.01) and PCG T1s (*Mean diff* = 1.34, *SE* = 0.59, *p* = 0.02).

#### Subjective Arousal

Arousal decreased across Tests [*F*(2,164) = 92.74, *p* = 0.0001, $\eta_{\text{p}}^{2}$ = 0.53]. No other main or interaction effect was significant.

#### Systolic Blood Pressure

Systolic blood pressure decreased over Tests [*F*(5,380) = 3.52, *p* = 0.004, $\eta_{\text{p}}^{2}$ = 0.04]. A significant main effect for Participants’ sex [*F*(1,76) = 13.74, *p* = 0.0001, $\eta_{\text{p}}^{2}$ = 0.15] showed that females had lower SBP (*mean diff* = –6.53, *SE* = 1.76, *p* = 0.0001). No other main or interaction effects were significant.

#### Diastolic Blood Pressure

Diastolic blood pressure decreased over Tests [*F*(5,72) = 2.52, *p* = 0.03, $\eta_{\text{p}}^{2}$ = 0.14]. No other main or interaction effects were significant.

Thus, except for the results for the subjective reports of stress, the results for the subjective arousal and physiological stress did not support our third and fourth hypotheses.

### Discussion

As in *Experiment One*, no placebo effects were seen on pain report in *Experiment Two* and thus, our first and second hypotheses were again not supported. However, there were findings, partially aligned with our fourth hypothesis, that showed effects of participant and experimenter sex on subjective and physiological stress levels. There was no placebo effect on pain reports in the PG T3. However, in the CCG T2 the reported pain was equivalent to the PCG T2, and in the PG T2 the reported pain was marginally lower than the PCG T2. There was a placebo response related to the sex of the experimenter, as female experimenters in the PG T3 received lower subjective stress compared to female experimenters in the PCG T3, partially supporting our fourth hypothesis. Regarding the other effects of the experimenter and participant sex, male experimenters received higher subjective stress and females had lower SBP than males. Moreover, female experimenters received higher expectations of cream effectiveness.

#### Pain Reports and Expectancy

The conditioning procedure along with the administration of the cream with suggestive information led to a marginal lower pain in the PG T2 as compared to the PCG T2. However, such conditioned lowered pain was not translated to T3, probably because the relief from pain in T2 was relatively weak, and thus, was not carried over to the T3. Such weak effect is probably due to the uncertainty in the verbal information given to the PG and the CCG participants, as the difference between the lowered pain in the PG T2 compared to the PCG T2 was marginally significant. The insufficiency of the conditioned response is also evidenced by the cream effectiveness ratings; the cream was rated less effective from T2 to T3 in the PG and the CCG, and there were no group differences in cream effectiveness between the CCG and the PG; this indicates that the information provided was not optimal in producing expectations about the cream. Moreover, the significant correlations between the pain intensity UR and CR in different intensity stimuli in all three groups support this conclusion. Prior studies have also shown that a conditioning-only procedure might fail to generate placebo responses (e.g., Carlino et al., 2015; Flaten et al., 2018; Rhudy et al., 2018). Thus, as the verbal information was not effective enough to elicit a placebo effect on pain, the conditioning fell short to be effective as well. Furthermore, the reduction of pain over tests in all groups suggest that participants habituated to the pain stimulations.

Pain ratings in the PCG T2 were not lower than the CCG T2, even though the stimuli administered in the CCG was higher. This suggests that the mere application of the cream in the CCG could have elicited a placebo response in this group. This is also evidenced by the significant correlations between the difference between the T1 and the T3 stimuli. This fits well with the findings in Flaten et al. (2018), similarly designed as the present study, where a placebo response in a cream control group was observed. The lowered pain in the CCG T2 might be due to subtle contextual factors acting as placebos (e.g., Levine and Gordon, 1984; Galer et al., 1997; Daniali and Flaten, 2019). Such contextual or interactive subtle cues might have acted as a placebo and generated a positive expectation about the application of the cream, as evidenced by significant predictive effect of the cream effectiveness for the lowered pain in T2. However, the placebo effect elicited in T2 was not carried over to the T3. This might be due to a similar reason as explained above: the verbal information given to participants in the CCG was analogously ambiguous and not suggestive enough. Therefore, as the verbal information was not suggestive enough to generate positive expectations, as evidenced by no group difference in the cream effectiveness ratings between the PG and the CCG, the placebo response elicited by subtle factors was similarly weak to be translated to the T3.

#### Physiological and Subjective Stress

##### Participant sex

Females had lower SBP than males. This is in line with previous studies reporting a main effect of sex on SBP (e.g., Aslaksen et al., 2014, 2015) and in contrast with those that did not confirm such an effect (Robertson et al., 1991; Roderigo et al., 2017).

##### Experimenter sex

Female experimenters in the PG and CCG received lower pain compared to the PCG. This is not a placebo response, as the difference was seen already in the T1. Also, male experimenters received higher subjective stress and female experimenters received higher expectations of cream effectiveness. There was a placebo effect for female experimenters in the PG as they received lower subjective stress in the PG T3 compared to the female experimenters of the PCG T3. This evidence partially supports our fourth hypothesis. Previous studies have shown that being tested by female experimenters may lead to lower pain reports in males (e.g., Levine and De Simone, 1991; Gijsbers and Nicholson, 2005; Aslaksen et al., 2007), however, the present finding shows that being tested by female experimenters can also lead to lower subjective stress as well.

## General Discussion

This study investigated the following four hypotheses: (a) compared to males, female participants should have higher placebo effects (i.e., lower pain reports); (b) participants should have higher placebo effect (i.e., lower pain report) to experimenters of the opposite sex. (c) Following the placebo manipulations, female participants should have lower subjective and physiological stress levels compared to males; and (d) following the placebo manipulations, participants should have lower subjective and physiological stress levels when being tested by female experimenters.

There were no placebo effects on pain in *Experiment One*, and in *Experiment Two*, the placebo effect was not translated to the T3. In *Experiment One*, the failure to observe a placebo effect on pain is probably justified by the design of the study. In *Experiment Two*, however, the ambiguity of the verbal suggestion halted the experience of relief from pain. Interestingly, there was a placebo effect in the CCG T2 that only received the cream without the surreptitiously lowering pain, which we assume is due to the effects of subtle contextual factors (e.g., Levine and Gordon, 1984). However, this effect was not translated to the T3. The verbal information schemed for *Experiment Two* was not designed to separately generate a positive expectation on its own, but only in combination with the conditioning procedure. Although some studies have successfully generated a placebo effect by a conditioning-only procedure (e.g., de Jong et al., 1996; Klinger et al., 2007), the present study did not (e.g., as in Flaten et al., 2018; Rhudy et al., 2018). The results did not support our first and second hypotheses in both experiments, however, there were other results in both experiments suggesting the effects of participant and experimenter sex on subjective and physiological stress that were partially in line with our third and fourth hypotheses.

### Placebo Effects in Both Experiments

There were effects of participant and experimenter sex on stress levels in both experiments. In *Experiment One*, males had lower DBP following the placebo manipulation. Moreover, the female experimenter in the PC recorded lower DBP compared to the female experimenter of the NHC. In *Experiment Two*, female experimenters received lower subjective stress in the PG T3 compared to the PCG T3. Thus, the conditioning procedures/placebo manipulations seem to have been successful in inducing placebo responses on subjective and physiological stress, but not on pain. Although not hypothesized originally, this finding may advocate a distinction between the underlying mechanisms responsible for generation of the placebo effect on pain and on stress (e.g., Flaten et al., 2006; Aslaksen and Flaten, 2008; Roderigo et al., 2017). This finding is also in consensus with neurological studies suggesting the involvement of distinctive, yet overlapping neural networks in the process of sensory, cognitive, and affective aspects of pain (e.g., Craggs et al., 2007; Wiech et al., 2008). For instance, Craggs et al. (2007) mapped the connectivity of five brain regions (the anterior and posterior insula, dorsal anterior cingulate cortex, dorsal prefrontal cortex, and supplementary motor area) known to be involved in processing of pain and placebo effects. They reported that although following a placebo manipulation (i.e., applying a saline gel introduced as lidocaine prior to a painful rectal distention) similar patterns and reductions in the network activities between the brain regions (e.g., input from anterior insula to dorsal anterior cingulate cortex) were observed in the left and the right hemispheres, there were also differences in the network connectivity pattern and amplitude of the neural activations between hemispheres (e.g., lower input from anterior insula to dorsal anterior cingulate cortex in the right hemisphere).

Males in *Experiment One* had lower DBP following the placebo manipulation. To the best of our knowledge only one study has reported a diminishing trend on DBP and a decrease on SBP following exposure to a sham treatment (Ghione et al., 2004). In that study, the effects of exposure to either an electromagnetic field or a sham exposure on pain and cardiac activity was investigated. The results showed although exposure to the sham electromagnetic field did not generate a placebo effect on pain, it significantly reduced SBP and generated a diminishing trend of DBP that was close to significance. Therefore, this is the first study reporting a placebo response on DBP, and thus, is opposing the former findings that did not analyze DBP data in placebo studies (e.g., Roderigo et al., 2017). This finding suggests an informative value for DBP in exploring the effects of placebo manipulations on the physiological stress levels associated with pain. However, it should be noted that BP, in general, is shown to be an unreliable placebo cardiac index (Daniali and Flaten, 2020). Moreover, these findings contrasting with our third hypothesis, suggest that lower DBP following the placebo manipulation is more likely to occur in males.

There were main effects for the sex of the experimenter. In *Experiment One*, the female experimenter in the PC recorded lower DBP compared to the female experimenter in the NHC. Also, in *Experiment Two* female experimenters received lower subjective stress in PG T3 compared to PCG T3. Moreover, female experimenters received lower pain reports and higher cream effectiveness expectations compared to male experimenters. These findings are in line with our fourth hypothesis. Subtle factors have been shown to affect placebo responses (Levine and Gordon, 1984; Galer et al., 1997). Along the same lines, Kaptchuk et al. (2008) has shown that the interactive factors such as non-verbal behavior of the experimenter, augments placebo responses. Although a script was used to convey the verbal information, the non-verbal behaviors of the experimenters, for instance, were not controlled for. Thus, possibly the longer time spent to convey the information in the PC in *Experiment One* and the PG and the CCG in *Experiment Two*, has facilitated a non-verbally richer interaction by the female experimenters that may have led the participants to perceive female experimenters as more friendly in both experiments.

## Recommendations for Future Studies

Firstly, the repeated measure design of *Experiment One* failed to induce a placebo effects on pain report, probably due to a short resting time interval between conditions and testing both conditions on the same day. Therefore, prospective studies with a repeated-measures design are recommended to avoid testing participants with short time intervals between conditions. Based on the findings from *Experiment Two*, the verbal instruction was not suggestive, and the conditioning failed to induce a placebo effect robust enough to carry over from the manipulation trial to the post-test. Secondly, the present study suggested a distinction between placebo effects on pain and on associated stress; this remains to be replicated by other studies. Moreover, the underlying neural networks of such distinction in the processing of sensory and affective aspects of pain and placebo remains yet to be better understood. Thirdly, this study showed that DBP can be informative in placebo investigations especially when the effects of placebo manipulation on cardiac stress is of focus. This finding is novel and needs to be replicated by other studies. Fourthly, it is concluded that subtle social and interactive factors can mediate the effects of sex in placebo contexts, however, this notion is not directly tested in this study. Although a few studies have investigated the effects of subtle social and interactive factors on enhancement of placebo effects (for a review see Daniali and Flaten, 2019), the interaction of such factors (e.g., the facial expression of experimenters) with the sex of the experimenter is yet to be fully discovered. Fifthly, the results suggested that being tested by female experimenters leads to lower subjective and physiological stress levels. Campbell et al. (2006) showed that the characteristics of experimenters (such as higher status) can mediate the participants’ blood pressure and pain report. Our study now adds that the female character can mediate placebo effects on subjective and physiological stress level. These findings should be replicated in prospective studies. Next, and contrary to what was hypothesized originally, the placebo effect on associated stress occurred in males, rather than females; this requires replication. Lastly, the effects of cognitive constructs such as beliefs, and also the role of personality traits (e.g., Daniali and Flaten, 2021) on manifestation of placebo or nocebo effects needs to be considered in future investigation.

## Limitations

The present study bears a number of limitations. First is the design of the *Experiment One*, that led to failure to observe a placebo effect on pain report. To overcome the shortcomings in *Experiment One*, *Experiment Two* was carried out, however, the verbal instructions and the conditioning procedures in *Experiment Two* failed to produce placebo effects on pain report. This is contrary to some studies that observed a conditioned placebo effect with implementation of a conditioning-only procedure (e.g., Klinger et al., 2007). Moreover, although there were placebo effects on pain-associated stress levels in both experiments, indicating the adequacy of the statistical power of the samples to detect the effects of placebo manipulations in both experiments, still the small blocks, specifically for the effects of participant and the experimenter sex may have reduced the statistical power for detection of placebo effects on pain. For instance, the sample size for *Experiment Two* was computed for a moderate to large effect size, based on the previous studies. However, assuming a smallest effect size of interest (Lakens et al., 2018), in our case, e.g., an effect size of 0.01, would have required a sample size over 200 participants. Second and fourth limitations concern BP measurements: in both experiments, the BP was measured only once in each measurement point. Although this method has been used successfully in former placebo studies (e.g., Robertson et al., 1991; Aslaksen et al., 2014, 2015; Geers et al., 2015; Roderigo et al., 2017), measuring BP for a single time on each measurement point can be subject to flaws. Instead, it is recommended to measure BP several times in each measurement point and use the average of the readings (e.g., Williams et al., 2018). Next is the white lab coat effect on BP measurements. It is demonstrated that being tested by a physician/experimenter transiently affects BP (Pickering et al., 2002). Therefore, although in both studies the placebo manipulation impacted BP measurements, it’s still possible that the BP readings were affected by the white lab coat phenomenon, as the assistants were present at the time of measurement. Therefore, prospective studies are recommended to either employ methods to measure the white lab coat effect or investigate the effects of placebo manipulations in a context free from factors that impose such effects. Last limitation concerns the lack of a proper control for subtle interactive factors in both experiments. However, as the effects of such subtle factors are being overlooked by most studies, this study alongside with more direct evidence (e.g., Chen et al., 2019; Daniali and Flaten, 2019) recommends controlling for the effects of such subtle factors.

## Conclusion

Several conclusions can be drawn from the present study: a conditioning-only procedure to generate a placebo effect on pain report is more likely to fail compared to a procedure including both the conditioning and rigid verbal suggestions; there is a distinction between mechanisms responsible to generate placebo responses on pain and on stress; that DBP data can be informative on indexing placebo-related changes on stress levels; and contrary to our third hypothesis, male participants can have lower DBP following a placebo manipulation. Lastly, and in line with our hypothesis, being tested by female experimenters may lead to lower pain reports and stress levels.

## Data Availability Statement

The raw data supporting the conclusions of this article will be made available by the authors, without undue reservation.

## Ethics Statement

The studies involving human participants were reviewed and approved by Regional Committee for Medical Research Ethics North Norway. The patients/participants provided their written informed consent to participate in this study.

## Author Contributions

SV and MF planned, designed, and conducted the experiments. HD and MF analyzed the data and structured the manuscript. All the authors then collaborated on drafting, revising, and preparing the final manuscript.

## Conflict of Interest

The authors declare that the research was conducted in the absence of any commercial or financial relationships that could be construed as a potential conflict of interest.
